# Retraction: An epigenetic change in a moth is generated by temperature and transmitted to many subsequent generations mediated by RNA

**DOI:** 10.1371/journal.pone.0360873

**Published:** 2026-10-20

**Authors:** The PLOS One Editors

Following the publication of this article [1], concerns were raised about Fig 1 and S1 Data. Specifically,

- The moths presented in Fig 1A and Fig 1B appear more similar than would be expected from independent specimens.
- Data presented in the Reversed tab of S1 Data appear more repetitive than would be expected.
- The article makes claims about epigenetic changes lasting 40 generations, but no data past the 12^th^ generation are provided with the article.

Regarding the concerns with Fig 1, the corresponding author stated that the images in this figure were assembled from black-and-white photographs and scanned material as an illustrative comparison intended to show the gross visual difference between the short- and long-antenna phenotypes. They commented that the images included did not originate from experimental image datasets and were not used for phenotype scoring, morphometric measurements, frequency calculations, statistical analysis, or any other quantitative result reported in the article. The corresponding author stated that they are unable to provide the raw, unprocessed acquisition files underlying Fig 1 or photographs of each phenotype. They provided photographs and SEM images from a different/related study, but PLOS does not consider that repeat experiment data are sufficient to resolve the concerns raised with Fig 1 in [1].

Regarding the dataset concerns with S1 Data, the corresponding author stated that although individual offspring were examined and phenotypically scored, the records were retained as sex- and phenotype-specific counts within each clutch, as opposed to a database assigning a unique identifier to every individual offspring. They provided the clutch-level count for the 233 clutches analyzed in generations F1-F3, F5, and F12 for editorial review. When providing these data, the corresponding author commented that 24 of the 233 clutches included during the analysis reported in the article were inadvertently omitted from S1 Data. Furthermore, they stated that they identified the following errors in S1 Data:

- Copied cell references that shifted during formula propagation.
- Omission of asymmetric individuals from the denominator.
- Incomplete summation of the sex-specific short-antenna counts.

The corresponding author stated that they recalculated all percentages directly from the recovered underlying phenotype counts and independently reanalyzed the complete set of 233 clutches. They stated that this resulted in a change of classification for one of the 233 clutches and provided the updated dataset to PLOS. Editorial review of the corrected dataset raised further concerns about the reliability of these data.

Regarding the concerns that no data past the 12^th^ generation are provided with the article, the article’s Materials and methods states, “Over 10–20 lines in each generation (always descendants of one pair) carrying epigenetic information were monitored for forty generations. We counted a proportion of epigenetic trait (saWT) and non-epigenetic trait (sa) for individuals in F1–F3, F5 and F12 generations. F4, F6–F11 and F13–F40 generations were just observed without precise counting of sa and sa^WT^ phenotype.” The *PLOS One* Editors remain concerned that results and conclusions are reported for 40 generations, when supporting data for these findings were only recorded up to the 12^th^ generation, and that in the absence of generation-by-generation underlying records for these observations, the central claim of persistence through F40 cannot be independently verified by the editorial team. The corresponding author maintains that the persistence of the phenotype was observed through F40, but accept PLOS’ concern that quantitative generation-by-generation records for F13–F40 are no longer available and that therefore these observations cannot now be independently verified. They offered to remove all evidentiary claims concerning persistence beyond F12 from the article, but PLOS considers that the claim of epigenetic changes lasting 40 generations was presented as one of the key findings of the study, and PLOS does not consider partial retractions.

In light of the above concerns, which call into question the reliability and integrity of the reported results, the *PLOS One* Editors retract this article.

PG agreed with the retraction. JP did not agree with the retraction. SP and VP either did not respond directly or could not be reached.
